# Nematodes immunomodulatory mechanisms and possible clinical application

**DOI:** 10.3389/fimmu.2026.1725730

**Published:** 2026-04-16

**Authors:** Nikodem Świderski, Mateusz Gumienny, Anna Bielecka-Wajdman

**Affiliations:** Department of Pharmacology, Medical University of Silesia, Katowice, Poland

**Keywords:** *Ancylostoma duodenale*, *Anisakis pegreffii*, *Ascaris lumbricoides*, *Brugia malayi*, cystatin, ES-62, helminth immunomodulation, immune tolerance

## Abstract

Helminths have evolved complex immunomodulatory mechanisms that enable chronic infection while minimizing host damage. This review summarizes recent advances in understanding nematode-derived molecules with therapeutic potential. The phosphorylcholine-modified glycoprotein ES-62 from *Acanthocheilonema viteae* exemplifies pathway-selective immune regulation and has inspired small-molecule analogues for inflammatory diseases. *Ancylostoma duodenale* secretes protease inhibitors, anticoagulants, and anti-inflammatory proteins that reveal targets for immune and hemostatic modulation. *Anisakis pegreffii* exerts context-dependent immune control via dendritic cell tolerization, macrophage polarization, and regulatory T-cell expansion. The cystatin Al-CPI from *Ascaris lumbricoides* reprograms dendritic cell metabolism, induces immune tolerance, and shows efficacy in colitis and allergy models. *Brugia malayi*secretes proteins that modulate antigen presentation, complement activation, and cytokine signaling. Together, these findings highlight helminth-derived compounds as blueprints for precision immunotherapies that restore immune homeostasis with minimal side effects.

## 1 Introduction

Helminths have co-evolved with their vertebrate hosts over millions of years, refining an array of molecular strategies that recalibrate rather than ablate host immunity. This evolutionary arms race has yielded secreted proteins, glycoconjugates, enzyme inhibitors, and extracellular vesicles that collectively promote parasite persistence while minimizing pathology (1). EVs, in particular, serve as key delivery systems for helminth-derived immunomodulatory molecules, including RNAs, proteins, and lipids that can induce trained immunity in host cells. For instance, EVs from nematodes like Heligmosomoides polygyrus carry miRNAs that suppress inflammatory responses and promote regulatory T-cell expansion, offering potential for therapeutic applications in inflammatory diseases (2). In recent years, these parasite-derived immunomodulators have attracted intense interest as blueprints for targeted therapies against chronic inflammatory and autoimmune diseases, offering the prospect of immune homeostasis with fewer off-target effects than broad immunosuppression.

Among filarial nematodes, the excretory–secretory glycoprotein ES-62 from Acanthocheilonema viteae has emerged as a benchmark molecule linking precise structural features—most notably phosphorylcholine-modified N-glycans—to systems-level immune reprogramming. ES-62 subverts canonical inflammatory signaling by selectively routing key transducers for autophagolysosomal degradation and resetting dendritic cell, B- and T-cell, macrophage, and mast-cell responses. The subsequent development of small-molecule analogues that reproduce ES-62’s pathway-selective modulation underscores the translational feasibility of helminth-inspired therapeutics (3, 4).

Convergent immunoregulatory themes extend beyond filariae. The human hookworm Ancylostoma duodenale deploys a coordinated arsenal of protease inhibitors, anticoagulants, anti-inflammatory proteins, and tissue-modifying enzymes to establish chronic, low-pathology infections in the intestinal niche. This repertoire not only illuminates mechanisms of immune evasion and mucosal adaptation but also points to novel candidates for modulating neutrophil activity, coagulation, and excessive inflammation (5, 6). Likewise, the zoonotic nematode Anisakis pegreffii exhibits sophisticated, context-dependent control of host responses—tolerizing dendritic cells, polarizing macrophages, expanding regulatory T cells, and tuning antigen expression in a temperature- and tissue-specific manner—highlighting both therapeutic promise and the need to manage strain variation and safety (7). The human roundworm Ascaris lumbricoides contributes a structurally and functionally well-defined cystatin (Al−CPI) that reprograms dendritic cell metabolism and phenotype, enhances regulatory circuits, and attenuates Th2-driven pathology, with efficacy across models of allergic airway disease and colitis and a favorable allergenicity profile (8). Finally, Brugia malayi provides a diverse secretome—including cystatin, galectin-2, calreticulin, IL−5 receptor binding protein, and abundant larval antigens—capable of reshaping antigen presentation, complement activation, eosinophil biology, and T-cell polarization; several of these factors already show promise as vaccine components, adjuvants, and disease-modifying agents in non-helminth models (9, 10).

Despite accumulating molecular detail, critical translational limitations persist. Most evidence derives from *in vitro* studies using human cells or animal models, with few helminth-derived immunomodulators advancing to clinical testing. Key concerns include immunogenicity of foreign proteins, batch-to-batch variability in complex biologics, endotoxin contamination in recombinant preparations, and potential safety issues from dual pro-/anti-inflammatory properties. First, the field needs a coherent, cross-species framework that maps helminth effectors to their cellular targets, signaling nodes, and biophysical dependencies, distinguishing context-appropriate immune rebalancing from blunt suppression. Second, translational strategies must bridge from complex glycoproteins and vesicles to stable, scalable biologics or small-molecule mimetics with well-defined pharmacology and safety. Third, rational clinical positioning—identifying indications where pathway-selective immunomodulation adds value over existing biologics—requires robust biomarkers of engagement and response. Foundational clinical evidence supports the immunoregulatory properties of natural helminth infections in autoimmune diseases. Well-tolerated natural helminth infections have been shown to protect against the worsening of multiple sclerosis (MS) symptoms in human (11, 12). More recently, natural helminth infections (including Strongyloides stercoralis, Hymenolepis nana, Enterobius vermicularis, Ancylostoma duodenale, Ascaris lumbricoides, and Trichuris trichiura) enhance the negative regulatory axis of TYRO3, AXL, and MERTK (TAM) receptors and their ligands in MS patients. This mechanism appears critical for controlling the inflammatory response by both reducing the proportion of the Th17 subset and downregulating its pathogenic genetic program (13). These findings underscore the translational potential of helminth-derived molecules in restoring immune homeostasis.

**Figure 1:**
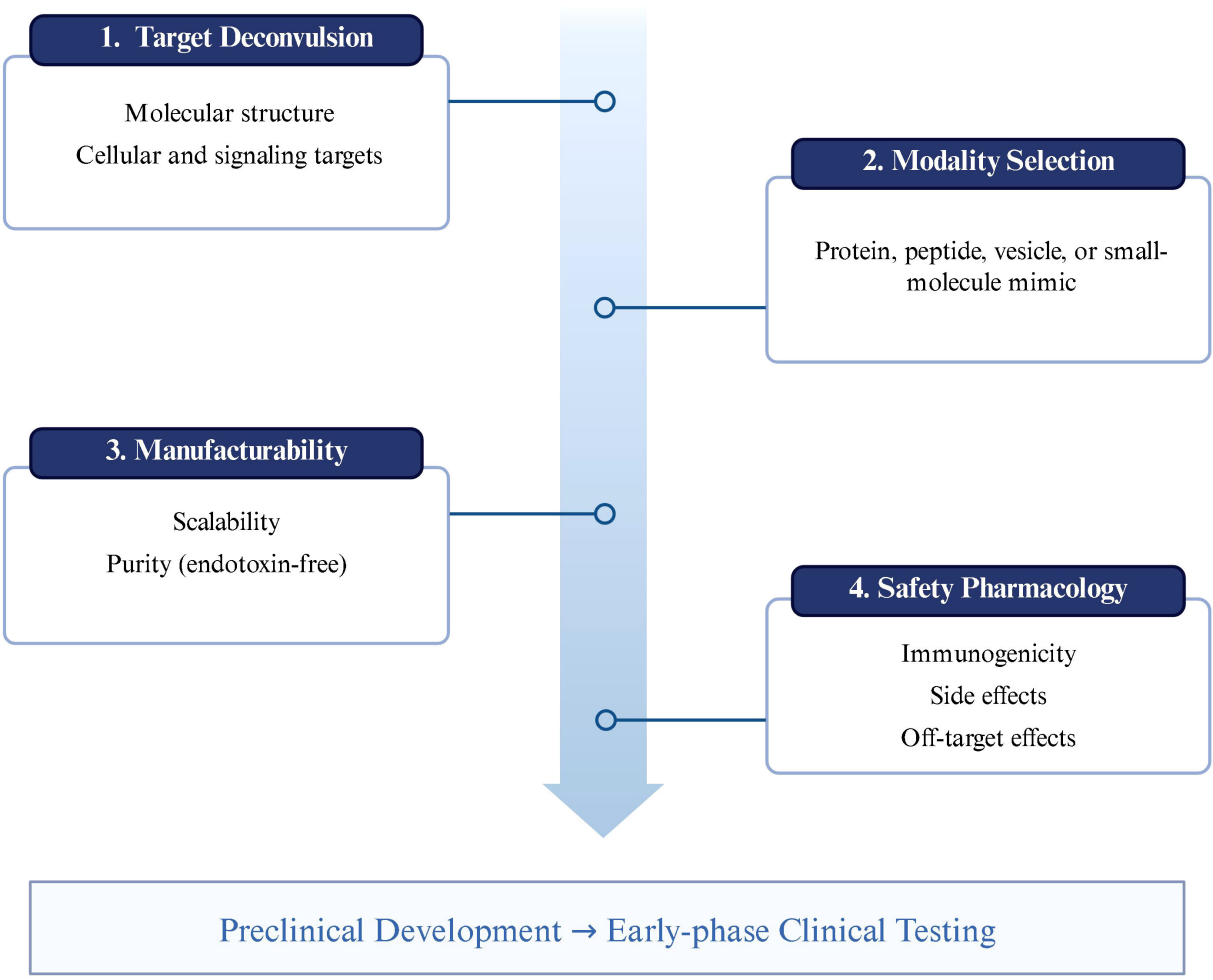
Roadmap.

This manuscript addresses these gaps by: (i) synthesizing structural and mechanistic insights on ES−62 and its synthetic analogues; (ii) delineating Ancylostoma duodenale’s immunomodulatory and hemostatic toolkits with therapeutic read-through; (iii) defining Anisakis pegreffii’s dual pro-/anti-inflammatory programming across dendritic cells, macrophages, and Tregs, including temperature-responsive antigen expression; (iv) consolidating evidence for Ascaris lumbricoides cystatin as a clinical candidate that couples dendritic cell metabolic rewiring to durable immune tolerance; and (v) surveying Brugia malayi’s immunoactive secretome spanning complement antagonism, cytokine receptor blockade, and vaccine-enabling antigens. We integrate these case studies into a translational roadmap emphasizing target deconvolution, modality selection (protein, peptide, vesicle, or small-molecule mimic), manufacturability, and safety pharmacology. **Figure 1** outlines a translational development framework for helminth-derived immunomodulators, beginning with target deconvolution and structural characterization, followed by modality selection (e.g., proteins, peptides, or small-molecule mimetics), manufacturability considerations, and safety pharmacology. This pipeline highlights key steps required to advance candidate molecules toward preclinical development and early-phase clinical testing.

By positioning helminth-derived molecules as precision immunomodulators that restore homeostasis rather than impose global suppression, this work outlines design principles for a new therapeutic class against chronic inflammatory and autoimmune diseases and identifies actionable priorities for preclinical development and early-phase clinical testing.

## 2 Materials and methods

This narrative review was conducted to synthesize current evidence on the immunomodulatory mechanisms of selected nematodes and their potential clinical applications. A systematic literature search was performed exclusively using the PubMed database for articles published in English from inception until October 2025. The search strategy employed a combination of Medical Subject Headings (MeSH) terms and keywords, including “ES-62”, “Acanthocheilonema viteae”, “Ancylostoma duodenale”, “Anisakis pegreffii”, “Ascaris lumbricoides”, “Brugia malayi”, “immunomodulation”, and “therapeutic application”. The titles and abstracts of identified records were screened for relevance, followed by a full-text assessment of selected articles. The study selection prioritized original research articles and authoritative reviews that provided mechanistic insights into nematode-derived immunomodulatory molecules. Given the heterogeneity of the subject, a narrative synthesis approach was adopted to thematically summarize the structural characteristics, mechanisms of action, and therapeutic potential of the identified molecules.

## 3 Acanthocheilonema viteae - ES-62

ES-62 represents one of the most extensively characterized excretory-secretory products derived from parasitic helminths, specifically the filarial nematode *Acanthocheilonema viteae* (4). This phosphorylcholine (PC)-containing glycoprotein has emerged as a paradigmatic example of how parasitic organisms have evolved sophisticated mechanisms to modulate host immune responses, not merely for survival, but potentially as a source of novel therapeutic interventions for inflammatory diseases (14). The molecule’s unique structural features and profound immunomodulatory properties have positioned it at the forefront of research into helminth-derived therapeutics, particularly in the context of autoimmune and allergic disorders.

ES-62 is a tetrameric glycoprotein with a molecular mass of approximately 240 kDa, comprising identical monomers of ~62 kDa, from which its nomenclature derives (4). Sedimentation equilibrium analytical ultracentrifugation has demonstrated that ES-62 exists as a tightly bound tetramer formed from dimers with a dissociation constant of 50 nM (File 4). The protein exhibits 37-39% sequence homology with a family of six other proteins, including murine and human aminopeptidases, suggesting potential enzymatic activity (4).

The most distinctive structural feature of ES-62 is its post-translational modification with phosphorylcholine, attached to one of three distinct N-linked glycans characterized on the molecule (4). Fast atom bombardment mass spectroscopy analysis revealed three types of N-glycan structures: high mannose type, a truncated form trimmed to the tri-mannosyl core with sub-stoichiometric fucosylation, and a trimannosyl core with or without core fucosylation carrying between one and four additional N-acetylglucosamine residues. The phosphorylcholine moiety is specifically attached to the third type of glycan and appears to be present in more than one copy per glycan (4).

The biosynthesis of phosphorylcholine-containing glycans occurs as a post-endoplasmic reticulum event, likely in the medial Golgi, and is dependent on mannosidase I but not mannosidase II activity. This unique post-translational modification is conserved among filarial nematodes and represents a potential target for chemotherapy, as these structures are absent from humans (4).

## 4 Immunomodulatory mechanisms

ES-62 exerts its immunomodulatory effects through multiple cellular targets within the immune system, including B lymphocytes, T lymphocytes, macrophages, and dendritic cells (4). The molecule’s interaction with these cells results in complex alterations to signal transduction pathways that collectively promote an anti-inflammatory phenotype. In B lymphocytes, ES-62 interferes with B cell receptor (BCR) signaling by selectively targeting downstream pathways coupled to BCR activation (14). The mechanism involves recruitment of SHP-1 tyrosine phosphatase to abolish BCR coupling to the Ras/Erk MAPK cascade by dephosphorylation of the Igα/β-Immunoreceptor Tyrosine-based Activation Motifs. Additionally, ES-62 recruits the ErkMAPK phosphatase Pac-1 and the negative regulator RasGAP, creating a multi-pronged mechanism that results in rapid desensitization of BCR signaling (14). Similarly, in T lymphocytes, ES-62 desensitizes T cell receptor (TCR) signaling by disrupting coupling to phospholipase D, protein kinase C, PI 3-kinase, and Ras-Erk MAPK signaling pathways. This desensitization occurs without affecting the generation of second messengers by phospholipase C, indicating selective targeting of specific signaling cascades (14).

Recent investigations have revealed that ES-62 modulates dendritic cell responses through a sophisticated mechanism involving the selective autophagolysosomal degradation of Toll-like receptor (TLR) transducers, exemplified by protein kinase C-δ (PKCδ) (15). This process represents a homeostatic regulatory mechanism that ES-62 has evolved to harness for limiting aberrant inflammation. ES-62 was prepared endotoxin-free by rigorous removal of LPS from Acanthocheilonema viteae culture supernatants, as confirmed by negative Limulus amebocyte lysate assays, and comparative controls using Salmonella minnesota or Escherichia coli 055:B5 LPS were included in parallel experiments. Autophagic flux kinetics were quantified by measuring the LC3-II/LC3-I ratio and p62 levels at 2, 6, 18 and 36 h after stimulation with ES-62 or LPS, demonstrating that ES-62 increases the LC3-II/LC3-I ratio—indicative of low-level autophagic flux—whereas LPS decreases this ratio, consistent with a block in autophagic flux. To verify the involvement of the autolysosomal pathway, the specific inhibitors E64d plus pepstatin A, NH_4_Cl and 3-methyladenine were employed; these agents prevented ES-62-induced degradation of p62, LC3 and PKCδ without affecting the response profile to LPS.

**Figure 2:**
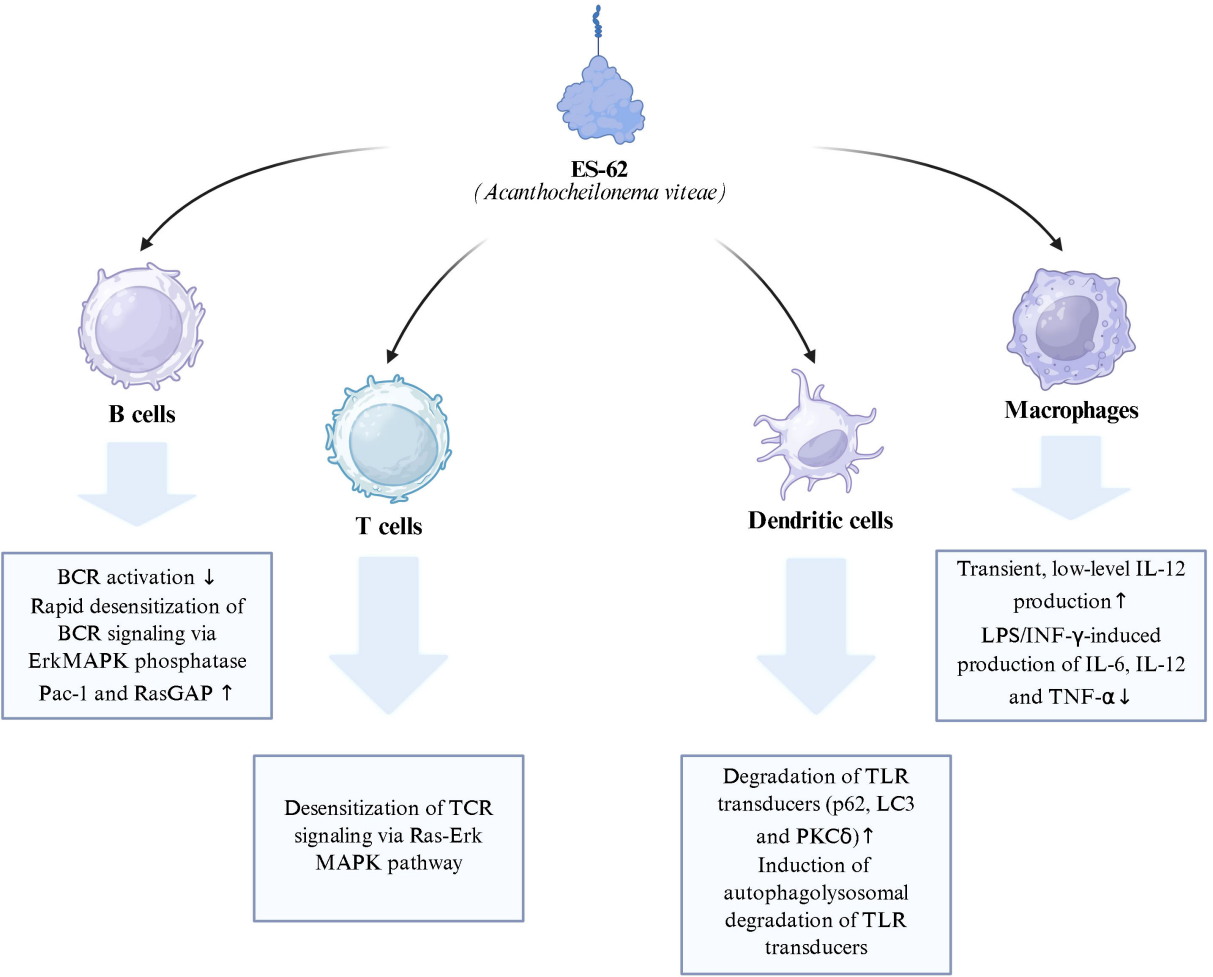
ES-62 mollecular mechanisms of action.

ES-62 treatment of dendritic cells results in the downregulation of PKCδ to approximately 50% of steady-state levels, which is functionally significant given PKCδ’s key roles in dendritic cell development, motility, IL-12p40/p70 production, and polarization of Th1 responses (15). This downregulation is achieved through induction of low-level autophagic flux, as evidenced by upregulation and trafficking of p62 and LC3 and their consequent autophagolysosomal degradation. In contrast to ES-62’s effects, the canonical TLR4 ligand lipopolysaccharide (LPS) strongly upregulates p62 and LC3 expression but appears to induce a block in autophagic flux, with these elements being predominantly degraded in a proteasomal manner (15). This differential handling of autophagy-related proteins distinguishes ES-62’s anti-inflammatory mechanism from classical pro-inflammatory TLR4 signaling. ES-62 utilizes TLR4 for its immunomodulatory actions, but unlike canonical TLR4 ligands, it does not signal for robust pro-inflammatory responses (15). The molecule requires TLR4 for its effects on dendritic cells, macrophages, and mast cells, but employs this receptor in a novel manner that subverts rather than activates classical inflammatory pathways (14). **Figure 2** integrates the cell-specific effects of ES-62, illustrating how TLR4-dependent signaling leads to selective autophagolysosomal degradation of PKCδ in dendritic cells, disruption of BCR and TCR signaling pathways, and induction of a hypo-responsive phenotype in macrophages and mast cells. This coordinated, multi-cellular modulation underlies its pathway-selective anti-inflammatory activity.

In macrophages, ES-62 exposure results in cells that are refractory to induction of pro-inflammatory responses by subsequent stimuli. While ES-62 can induce low-level and transient production of IL-12, it blocks the subsequent substantial production of this cytokine, as well as IL-6 and TNF-α, when cells are challenged with LPS/IFN-γ (3). This effect extends beyond TLR4 signaling, as ES-62-primed macrophages show suppressed responses to TLR2 and TLR9 ligands, indicating that TLR4 engagement by ES-62 enables cross-desensitization of multiple TLR pathways (14). These macrophages cell–specific effects form part of the broader ES-62 signaling network depicted in **Figure 2**.

In mast cells, ES-62 demonstrates the ability to desensitize FcϵRI-mediated responses through TLR4-dependent mechanisms (14). The molecule does not affect FcϵRI expression but inhibits FcϵRI-triggered degranulation by downregulating PKC-α through caveolae/lipid raft-mediated endocytosis of ES-62/TLR4/PKC-α complexes. This sequestration and degradation of PKC-α disrupts coupling of FcϵRI to key PLD-SPHK-1-dependent pathways of calcium, PKC, and ultimately NF-κB activation (14).

## 5 Therapeutic applications

ES-62, a phosphorylcholine-modified glycoprotein from Acanthocheilonema viteae, exhibits strong therapeutic efficacy in collagen-induced arthritis models, significantly reducing disease severity even after onset. Its anti-arthritic effects involve suppression of IFN-γ, TNF-α, and IL-6, alongside increased IL-10 production and protection against joint damage. ES-62 also modulates the IL-23/IL-17/IL-22 axis via MyD88 downregulation, restoring immune homeostasis without broad immunosuppression. Synthetic small-molecule analogues, such as compound 11a, reproduce these effects with reduced immunogenicity and production cost. The phosphorylcholine moiety represents the key active component, defining ES-62’s unique immunomodulatory profile and therapeutic potential. The most extensively documented therapeutic application of ES-62 is in experimental models of rheumatoid arthritis. In the collagen-induced arthritis (CIA) model in DBA/1 mice, ES-62 significantly suppresses disease severity when administered during type II collagen priming and intraperitoneal challenge (3). Importantly, ES-62 demonstrates therapeutic efficacy even when treatment is initiated after disease onset, showing significant reduction of arthritis progression within 3 days of treatment commencement.

The anti-arthritic effects of ES-62 are mediated through suppression of collagen-specific Th1 responses, evidenced by significantly reduced IFN-γ, TNF-α, and IL-6 release in draining lymph nodes, while IL-10 production is increased (3). Histological evaluation reveals that ES-62 prevents progression of synovial hyperplasia and cartilage and bone erosion, indicating protection against articular damage (3). The therapeutic potential extends to human disease, as ES-62 can modify pro-inflammatory cytokine production in cells from rheumatoid arthritis patients. Primary cultures from rheumatoid arthritis synovial fluid and membrane show significant suppression of LPS-induced TNF-α and IL-6 in the presence of ES-62, demonstrating direct relevance to human pathology (3).

ES-62’s protective effects in inflammatory disease models are associated with homeostatic resetting of the IL-23/IL-17/IL-22 inflammatory axis, which becomes dysregulated in various inflammatory disorders. The molecule targets this axis through modulation of complex cellular interactions among dendritic cells, T cells, and γδ T cells, involving downregulation of MyD88 (14). Unlike neutralizing antibodies used clinically, ES-62 does not eliminate all IL-17 from the system but rather modulates its production in a context-dependent mannerc (14). In the CIA model, ES-62 downregulates Th1 responses, while in ovalbumin-induced airway hypersensitivity, it upregulates IFN-γ production, leading to downregulation of pathogenic IL-17 responses through cytokine cross-regulation (14).

Recognition of ES-62’s therapeutic potential has led to the development of synthetic small molecule analogues (SMAs) based on the phosphorylcholine moiety. These compounds offer several advantages over the parent molecule, including reduced immunogenicity, lower production costs, and the avoidance of potential interactions with natural anti-phosphorylcholine antibodies (14).

A sulfone-containing phosphorylcholine analogue, designated 11a, has demonstrated the ability to downregulate p65 NF-κB activation and inhibit IL-12p40 and IL-6 production in response to TLR2, TLR4, and TLR9 stimulation (14). In the CIA model, compound 11a proved as effective as ES-62 in protecting mice against arthritis, with protection associated with downregulation of IL-17/IFN-γ-dependent responses and inhibition of MyD88 expression (14). The immunomodulatory activities of ES-62 are predominantly attributed to its phosphorylcholine modification rather than its protein backbone. Phosphorylcholine conjugated to ovalbumin or bovine serum albumin can protect mice against CIA in a manner similar to ES-62, reproducing the expected downregulation of IL-17 without compensatory Th2 cytokine increases (14).

Comparative studies using recombinant ES-62 produced in yeast, which lacks phosphorylcholine, failed to reduce CIA disease severity or pro-inflammatory cytokine levels in joints, confirming the critical role of this post-translational modification (14). However, phosphorylcholine-conjugated proteins cannot fully replicate all ES-62 effects, such as alterations in anti-collagen IgG2a antibodies, indicating some contribution from the protein backbone or attached glycans (14).

## 6 Clinical implications and future directions

The body of research on ES-62 provides compelling evidence for the therapeutic potential of helminth-derived immunomodulators in treating chronic inflammatory diseases. The molecule’s ability to harness homeostatic regulatory mechanisms, such as autophagy-dependent degradation of pro-inflammatory signaling elements, offers a novel approach to immune intervention that differs fundamentally from current therapeutic strategies (15).

The development of small molecule analogues represents a significant advancement toward clinical translation, addressing the practical limitations of using a large, immunogenic protein as a therapeutic agent (14). The demonstration that structurally simple compounds can recapitulate ES-62’s anti-inflammatory properties opens new avenues for drug development based on helminth-derived immunomodulators.

## 7 Immunobiology of *Ancylostoma duodenale* and others

*Ancylostoma duodenale* represents one of the most significant soil-transmitted helminths affecting human health, with hookworm infections contributing substantially to neglected tropical diseases globally (16). The parasite’s remarkable ability to establish chronic infections lasting several years reflects sophisticated immunomodulatory mechanisms that have evolved to circumvent host immune responses while minimizing tissue damage (1). Recent advances in genomic analysis and molecular characterization have revealed the complex arsenal of immunomodulatory molecules that *A. duodenale* employs to maintain its parasitic lifestyle, presenting both challenges for parasite control and opportunities for therapeutic exploitation.

## 8 Immunomodulatory and invasion mechanisms

*A. duodenale* has developed sophisticated protease inhibition systems that play crucial roles in immune evasion and host tissue protection. The parasite secretes AduTIL-1, a serine protease inhibitor containing two trypsin inhibitor-like (TIL) domains that effectively inhibits human neutrophil elastase and pancreatic trypsin (6). This dual-domain structure allows AduTIL-1 to simultaneously target multiple host proteolytic systems, with the first TIL domain showing particularly strong inhibitory activity against human neutrophil elastase (Ki=114.9 nM) and bovine pancreatic chymotrypsin (Ki=18.0 nM), while the second domain demonstrates potent inhibition of human pancreatic trypsin (Ki=7.1 nM) (6). These Ki values were determined using recombinant domains expressed in Escherichia coli, with enzymes pre-incubated with inhibitors for 15 min at 25 °C in assay buffer, followed by chromogenic substrate addition for kinetic measurement at 405 nm.

The localization of AduTIL-1 in the esophagus, intestine, and cuticular surface of adult worms suggests multiple functional roles in parasite survival. By inhibiting neutrophil elastase, AduTIL-1 likely prevents host inflammatory responses and tissue damage at attachment sites, while its anti-trypsin activity protects the parasite from digestive enzyme damage within the host intestine (6). This multi-target approach represents an elegant evolutionary solution to the challenges of chronic parasitism in the digestive tract. The relationship between *A. duodenale* and host neutrophils represents a complex interplay of recruitment, activation, and evasion mechanisms. While neutrophils are increasingly recognized as important effector cells against hookworm larvae through the formation of neutrophil extracellular traps (NETs), *A. duodenale* has evolved sophisticated countermeasures to evade this host defense mechanism. The parasite actively evades NETosis through multiple pathways, including the secretion of DNase-II enzymes capable of degrading NETs, effectively preventing larval trapping and cuticle damage (17). These evasion mechanisms, such as DNase-II secretion, are largely extrapolated from closely related species like A. ceylanicum, A. caninum, and Necator americanus, drawing on conserved genetic features within Clade V nematodes; direct experimental evidence specific to A. duodenale remains limited.

Clinical evidence suggests that *A. duodenale* infections are associated with complex neutrophil responses that vary depending on the stage of infection and parasite load. The parasite’s ability to modulate neutrophil recruitment and function may contribute to its capacity for establishing chronic infections without triggering excessive inflammatory responses that could compromise both host and parasite survival (17). These observations are partly derived from zoonotic infections involving A. caninum and A. braziliense, as well as broader studies on soil-transmitted helminths, with specific data on neutrophil modulation in A. duodenale being indirect and warranting further investigation.

A. *duodenale* demonstrates remarkable sophistication in manipulating host hemostatic systems to facilitate blood feeding. The parasite secretes multiple anticoagulant factors, including AduNAP4, a unique anticoagulant peptide that inhibits both coagulation factor Xa (Ki = 7.34 ± 1.74 nM) and factor XIa (Ki = 42.45 ± 3.25 nM). This dual inhibition is particularly significant, as factor XIa plays a crucial role in amplifying the coagulation cascade through positive feedback mechanisms, making AduNAP4 an exceptionally effective anticoagulant (5). Factor XIa serves as a critical amplification enzyme in hemostasis, activated by thrombin on activated platelet surfaces to generate additional factor IXa, which supplements the initial activation pathway and sustains thrombin production through positive feedback loops. Clinically, factor XIa represents an attractive anticoagulant target because patients with congenital factor XI deficiency experience reduced thrombotic risk with minimal bleeding complications, suggesting that factor XIa inhibition may provide safer anticoagulation than traditional approaches targeting more essential hemostatic factors. These Ki values were determined using recombinant AduNAP4 expressed as a thioredoxin fusion protein in Escherichia coli, with enzymes (1 nM final concentration) incubated with inhibitors for 15 min at 25 °C in HBSAC buffer (50 mM HEPES, pH 7.4, 200 mM NaCl, 10 mM CaCl2, 0.2% bovine serum albumin, 0.1% NaN3), followed by chromogenic substrate addition for kinetic measurement at 405 nm.

Beyond specific factor inhibition, A. *duodenale* also secretes a 36,000 dalton proteolytic enzyme with both fibrinogenolytic and fibrinolytic properties. This protease not only prevents blood clot formation by degrading fibrinogen into non-clottable derivatives but also promotes the dissolution of existing fibrin clots, ensuring continuous blood flow for parasite feeding (18). The enzyme’s ability to convert plasminogen to mini-plasminogen-like fragments further enhances its fibrinolytic activity, creating an optimal environment for sustained blood feeding (18).

The initial stages of *A. duodenale* infection require sophisticated mechanisms for tissue penetration and migration. The parasite produces hyaluronidase enzymes that facilitate passage through host tissues during larval invasion. These enzymes, existing in both 87-kDa and 49-kDa forms, demonstrate substrate specificity for hyaluronic acid while showing no activity against chondroitin sulfate, distinguishing them from mammalian hyaluronidases and suggesting specialized evolutionary adaptations for parasitic invasion (19). The hyaluronidase activity is particularly pronounced in *A. duodenale* compared to related species, with enzyme activities reaching up to 3.3 μg of hyaluronic acid hydrolyzed per hour per μg of total parasite protein (19). This enhanced enzymatic activity correlates with the parasite’s ability to migrate extensively through epidermal and dermal tissues, contributing to its pathogenic potential in cutaneous larva migrans.

A concise overview of the best−characterized immunomodulatory and hemostatic mechanisms described for Ancylostoma species is presented in **Table 1**.

**Table 1:** Overview of immunomodulatory mechanism of Ancylostoma species.

Species	Molecule/mechanism	Target	Target cell/system	Model disease/context	Outcome
A. duodenale (6)	AduTIL-1 (serine protease inhibitor)	Human neutrophil elastase, pancreatic trypsin, bovine pancreatic chymotrypsin	Host proteolytic systems	Chronic parasitism in digestive tract	Prevents host inflammatory responses and tissue damage; protects parasite from digestive enzymes.
A. ceylanicum, A. caninum (17)	DNase-II enzymes	Neutrophil extracellular traps (NETs)	Neutrophils	Larval infection	Degrades NETs, preventing larval trapping and cuticle damage, allowing immune evasion.
A. duodenale (18)	AduNAP4 (anticoagulant petide)	Coagulation factor Xa, coagulation factor XIa	Host hemostatic system	Blood feeding	Inhibits the coagulation cascade, ensuring continuous blood flow for feeding.
A. duodenale (18)	36,000 dalton proteolytic enzyme	Fibrinogen and fibrin	Host hemostatic system	Blood feeding	Prevents blood clot formation by degrading fibrinogen and promotes clot dissolution by degrading fibrin.
A. duodenale (19)	Hyaluronidase enzymes (87-kDa and 49-kDa forms)	Hyaluronic acid	Host tissues (epidermal and dermal)	Larval invasion, cutaneous larva migrans	Facilitates tissue penetration and migration by degrading the extracellular matrix.

## 9 Possible therapeutic applications

**Figure 3:**
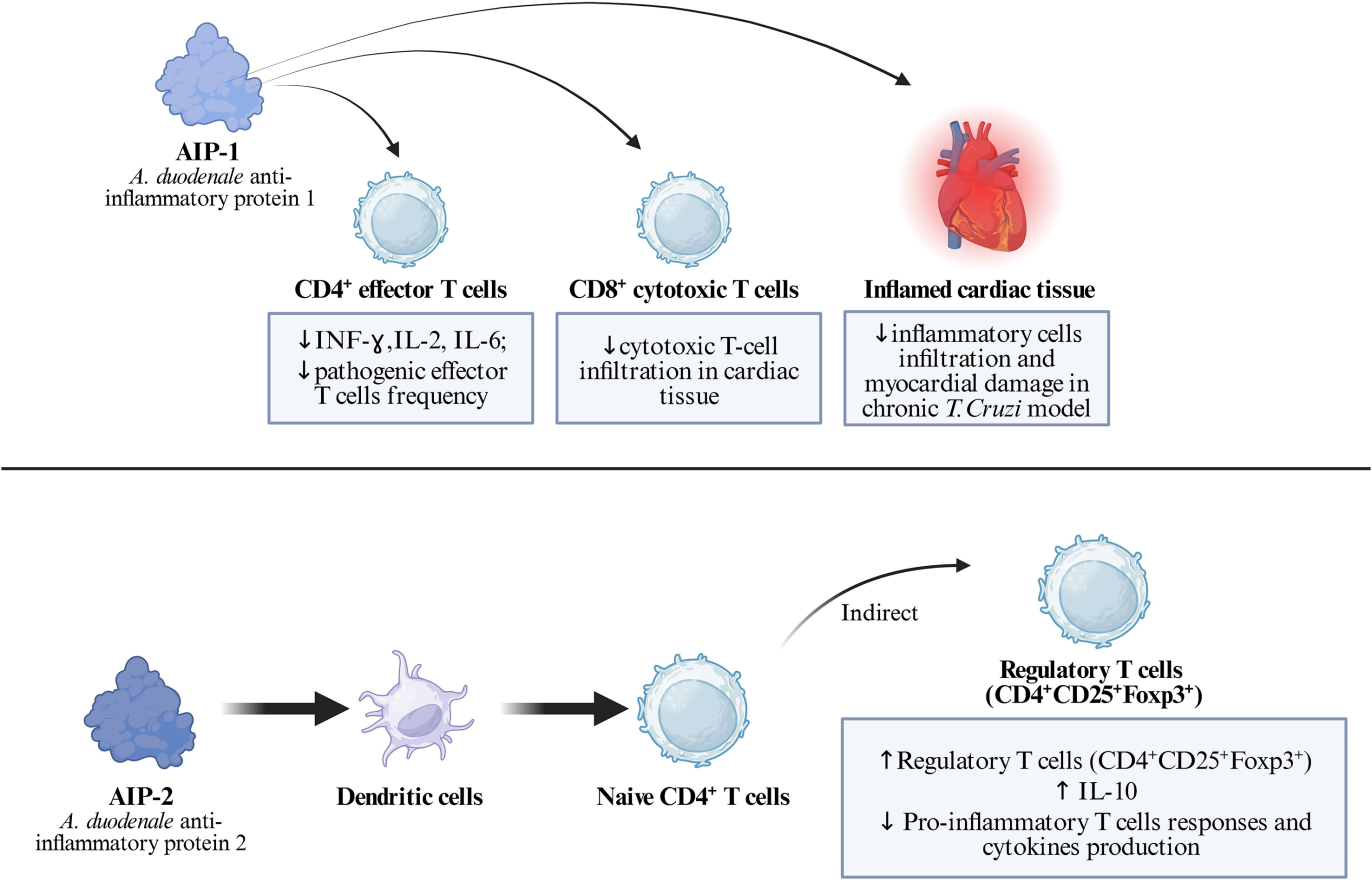
AIP 1 and AIP 2 immunomodulatory properties.

Recent research has identified hookworm-derived anti-inflammatory proteins with significant therapeutic potential. AIP-1 and AIP-2 (Anti-Inflammatory Proteins 1 and 2) represent promising therapeutic candidates for treating inflammatory. These proteins demonstrate remarkable efficacy in reducing cardiac inflammation in experimental models, with both AIP-1 and AIP-2 significantly reducing cellular infiltration and inflammatory cytokine levels including IFN-γ, IL-2, and IL-6 (20). The mechanisms underlying the anti-inflammatory effects of these proteins involve complex modulation of immune cell phenotypes and cytokine production. AIP-1 treatment specifically induces reduced levels of cytotoxic and pro-inflammatory T cells, while AIP-2 stimulation enhances regulatory T cell populations, suggesting distinct but complementary pathways for immune modulation (20). These findings support the broader concept that helminth-derived immunomodulatory proteins can serve as templates for developing novel anti-inflammatory therapeutics. **Figure 3** illustrates the distinct immunological effects of AIP-1 and AIP-2, highlighting their differential impact on T cell subsets, with AIP-1 primarily suppressing pro-inflammatory responses and AIP-2 promoting regulatory T cell expansion.

Advanced genomic analysis has provided unprecedented insights into the molecular basis of *A. duodenale*’s immunomodulatory capabilities. The recently completed chromosome-contiguous genome reveals a comprehensive secretome containing 3,171 excretory/secretory (ES) proteins, with significant enrichment in pathways related to metabolism, membrane trafficking, peptidases and inhibitors, and immune modulation (16). This diverse repertoire of secreted proteins represents the molecular foundation for the parasite’s sophisticated host manipulation strategies. The genomic analysis has identified conserved single-copy genes that lack detectable homologs in the human genome, representing potential targets for selective intervention. Among these, several encode secreted proteins with predicted immunomodulatory functions, including proteins involved in gap junction channel activity, oxidative phosphorylation, and thermogenesis (16). These parasite-specific molecular targets offer opportunities for developing selective therapeutic interventions that could disrupt parasite function without affecting host biology.

## 10 Clinical potential

The immunomodulatory mechanisms employed by *A. duodenale* have generated substantial interest for therapeutic applications in treating inflammatory and autoimmune diseases. The hygiene hypothesis suggests that reduced exposure to helminths in developed countries may contribute to increased rates of allergic and autoimmune diseases, positioning helminth-derived therapeutics as potential treatments for these conditions. Clinical trials using controlled helminth infections have shown mixed results in treating inflammatory bowel disease and other autoimmune conditions. While some studies with Trichuris suis demonstrated symptom improvement in subsets of inflammatory bowel disease patients, randomized controlled trials have yielded inconsistent outcomes, indicating that helminth-derived therapeutic effects may not be universal and require careful patient stratification (1). The development of recombinant versions of *A. duodenale* immunomodulatory proteins, such as AIP-1 and AIP-2, represents a promising approach for harnessing the therapeutic potential of helminth immunomodulation while avoiding the risks associated with live parasite infections (20). These proteins could serve as templates for developing novel anti-inflammatory drugs with enhanced specificity and reduced side effects compared to conventional immunosuppressive therapies.

## 11 Anisakis pegreffii

*Anisakis pegreffii*, a zoonotic marine nematode parasite, represents a significant public health concern through consumption of raw or undercooked seafood. Beyond its pathogenic role in human anisakiasis, emerging research reveals sophisticated immunomodulatory properties that present both therapeutic opportunities and complex host-parasite interaction mechanisms.

## 12 Dendritic cell modulation and tolerogenic mechanisms

A. pegreffii demonstrates profound immunomodulatory effects on human dendritic cells through multiple mechanisms. Live larvae significantly impact DC viability, reducing live cell percentages from 92% ± 8% (control) to 40% ± 25% in immature DCs and from 95% ± 2% to 55% ± 12% in mature DCs (7). The parasite impairs DC maturation by downregulating critical surface molecules including HLA-DR, CD86, CD83, and CCR7, while simultaneously increasing phagosomal reactive oxygen species (ROS) levels.

**Figure 4:**
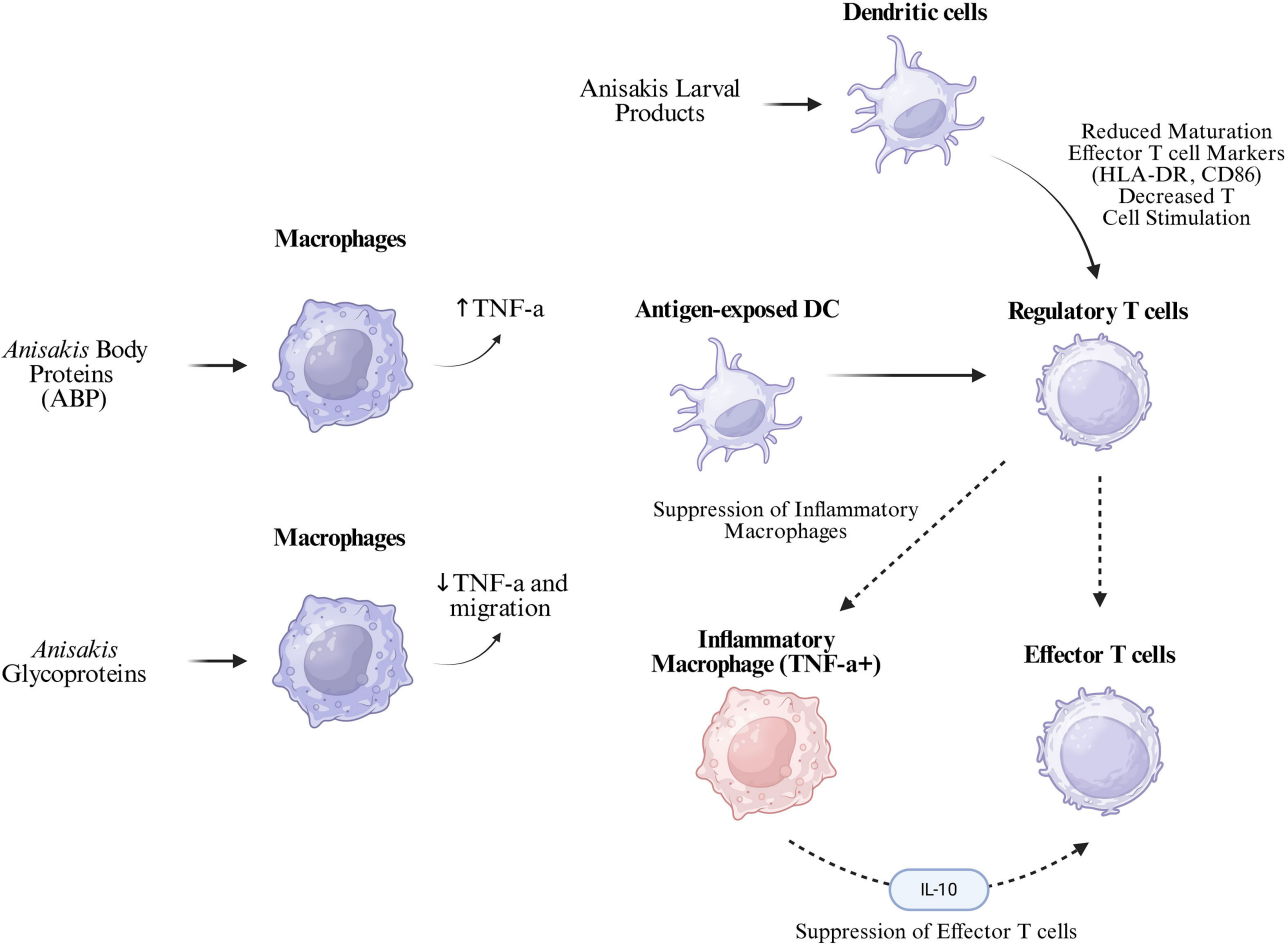
Immunomodulation properties of *A. pegreffii*. Adapted from: Zeng MH, Alsobaie S, Wang XX, Li S, Aldakheel FM, Aboul-Soud MAM, et al. Dual roles of *Anisakis pegreffii* proteins in macrophage immune dynamics. Front Immunol. 2025;16.

The molecular mechanisms involve modulation of the ERK1/2 signaling pathway, where mature DCs exposed to live parasites show strong decreases in ERK1/2 phosphorylation compared to controls, while NF-κB activation remains unaltered (7). The p62/SQSTM1 protein serves as both an autophagy receptor and a marker of autophagic flux, being rapidly degraded during autophagy activation. MG132, a proteasome inhibitor, is routinely used as a control to distinguish autophagy-mediated degradation from proteasome-mediated degradation, as it blocks proteasomal protein degradation while potentially inducing compensatory autophagy. This selective pathway modulation contributes to the parasite’s immune evasion strategy. *A. pegreffii* differentially modulates DC cytokine production patterns. Live larvae significantly downregulate CXCL10, CCL4 chemokines, and sICAM expression, while upregulating CCL3 chemokine production. Pro-inflammatory cytokines IL-6 and IL-1α show significant increases, particularly in immature DCs exposed to both live larvae and crude extracts (7). This complex cytokine modulation creates a microenvironment that favors immune tolerance while maintaining controlled inflammation. The dendritic cell–centered mechanisms by which. As illustrated in **Figure 4**, A. pegreffii disrupts dendritic cell maturation and cytokine signaling, promoting a tolerogenic phenotype.

The functional consequences of DC modulation manifest as impaired T-cell responses. DCs exposed to *A. pegreffii* show reduced capacity to activate T-cell-mediated IFN-γ production, contributing to the parasite’s ability to evade adaptive immune responses (7). This represents a sophisticated mechanism for immune evasion that allows persistent infection.

## 13 Macrophage polarization and dual immune response

Recent transcriptomic analysis reveals *A*. *pegreffii* induces contrasting immune responses through different molecular components. Anisakis body proteins (ABP) significantly enhance pro-inflammatory responses, with TNF-α showing a 1.83-fold increase compared to controls, reaching 1.97-fold when combined with LPS (21). This response involves activation of TNF signaling pathways and hematopoietic cell lineage pathways. Conversely, *A. pegreffii* glycoproteins demonstrate potent immunosuppressive properties. These glycoproteins significantly suppress TNF-α transcription to levels below control conditions (0.54-fold change) and reduce IL-4-induced Arg-1 expression (21). The mechanism involves enrichment of cytokine-cytokine receptor interactions and impaired leukocyte migration through downregulation of Ccl2 and H3c7.

**Figure 4** further demonstrates the dual macrophage response induced by A. pegreffii, with pro-inflammatory activation driven by body proteins and immunosuppression mediated by glycoproteins.

Protein-protein interaction network analysis identifies IL-6 as a central hub gene with 49 node degrees in ABP-treated samples, indicating extensive inflammatory network activation. In contrast, glycoprotein treatment shows only IL-1β, Ccl2, and H3c7 as major network nodes, with Ccl2 and H3c7 being downregulated (21). This differential gene expression pattern confirms the dual nature of *A. pegreffii*’s immunomodulatory arsenal.

## 14 Regulatory T cell expansion mechanisms

*A. pegreffii* larval antigens demonstrate sophisticated regulatory T cell expansion mechanisms that vary by host genetic background. In C57BL/6J mice, crude extract (CE)-stimulated dendritic cells significantly increase CD4+CD25-Foxp3+ and CD8+CD25-Foxp3+ populations, while excretory-secretory (ES) products show opposite effects (22). The expansion and functional profile of these regulatory T-cell subsets, in the context of A. pegreffii exposure, are summarized in **Figure 3**. This strain-specific response indicates host genetic factors influence the parasite’s immunomodulatory effectiveness. In BALB/c mice, CE-stimulated DCs cause expansion of CD4+CD25+Foxp3+IL-10+ and CD8+CD25+Foxp3+IL-10+ cells, while ES-stimulated DCs increase CD4+CD25+Foxp3+ and CD8+CD25-Foxp3+ expression (22). The accompanying IL-10 production from these regulatory populations creates a suppressive microenvironment that facilitates parasite survival. The regulatory T cells induced by *A. pegreffii* produce both IL-10 and IFN-γ, creating a unique immunoregulatory profile. IFN-γ expression increases primarily in CD4+CD25- and CD8+CD25- T cells after CE incubation in C57BL/6J mice, and in CD4+CD25+ T cells stimulated with CE and CD4+CD25- T cells stimulated with ES in BALB/c mice (22). This dual cytokine production represents a sophisticated balance between immune suppression and controlled inflammation. The downstream expansion of regulatory T cells and their cytokine profile are summarized in **Figure 4**.

*A. pegreffii* exhibits sophisticated temperature-dependent regulation of immunologically relevant proteins. The Kunitz-type trypsin inhibitor (A.peg-1) shows significant upregulation at both 20 °C (p = 0.026) and 37 °C (p < 0.0001) compared to 2 °C controls, with maximum expression at 37 °C representing homeothermic host conditions (23). Myoglobin (A.peg-13) demonstrates temperature-responsive expression with significant increases at 7 °C (p = 0.0092), 20 °C (p = 0.0292), and maximum expression at 37 °C (p = 0.0004) (23). Notably, the glycoprotein A.peg-7 shows no significant temperature-dependent modulation (ANOVA p = 0.7111), suggesting its regulation depends on other host factors such as immune responses rather than temperature. These expression profiles were determined using qRT-PCR on cDNA from larvae (n=3 biological replicates per temperature condition), with data normalized to GAPDH and analyzed by one-way ANOVA followed by unpaired Student’s t-test (no multiple comparison correction applied). The 37 °C condition mimics homeothermic hosts (definitive cetacean or accidental human), while 7–20 °C represent post-host conditions (7 °C as cold stress post-mortem of fish host, 20 °C as average ectothermic fish host temperature).

SDS-PAGE analysis confirms that gene expression patterns correlate with protein production. At 37 °C, all three target excretory-secretory proteins are detectable, while only A.peg-13 (37 kDa) is observable at 7 °C (23). This temperature-dependent protein production provides the parasite with adaptive mechanisms optimized for different host environments.

## 15 Therapeutic implications and applications

The demonstrated immunosuppressive properties of *A. pegreffii* glycoproteins present therapeutic opportunities. Their ability to suppress TNF-α transcription and reduce inflammatory cytokine production suggests potential development as anti-inflammatory agents for autoimmune diseases (21). The parasite’s capacity to induce functional regulatory T cells that produce both IL-10 and IFN-γ indicates potential applications in transplant immunology and autoimmune disease treatment. The dual cytokine profile creates unique immunoregulatory environments that could be therapeutically beneficial (22). Temperature-responsive antigen expression patterns suggest potential applications as vaccine adjuvants, where controlled inflammatory responses could enhance immune memory while preventing excessive inflammation (23).

## 16 Ascaris lumbricoides

Commonly known as the human roundworm, represents one of the most prevalent soil-transmitted helminth infections worldwide, affecting approximately 1.5 billion people globally (8). As a highly successful parasite, *A. lumbricoides* has evolved sophisticated mechanisms to modulate host immune responses, creating an anti-inflammatory environment that promotes parasite survival while simultaneously influencing the pathophysiology of other immunological conditions. The complex relationship between ascariasis and human health has garnered significant research attention, particularly regarding the parasite’s dual role as both a potential risk factor for allergic diseases and a source of immunomodulatory compounds with therapeutic potential.

## 17 Structural and biochemical characteristics of *A. lumbricoides* cystatin

*A. lumbricoides* produces a 14.2 kDa cysteine protease inhibitor (Al-CPI) that exhibits typical structural features of type-2 cystatins (24). The crystal structure of Al-CPI, determined at 2.1 Å resolution, reveals a conventional cystatin fold consisting of a five-stranded anti-parallel β-sheet wrapped around a central α-helix (25). This structure includes two conserved intramolecular disulfide bonds and contains key structural motifs responsible for protease binding activity, specifically loop 1 and loop 2 regions. The functional activity of Al-CPI has been extensively characterized, demonstrating strong inhibitory effects against cathepsin L, C, and S, with weaker inhibition of cathepsin B. These proteolytic activities are crucial for the molecule’s immunomodulatory functions, as cathepsins play important roles in antigen processing and presentation via MHC-II molecules (25). The specificity of Al-CPI for different cathepsins appears to be influenced by structural features, particularly the interaction between the inhibitor’s loop regions and the active sites of target proteases (25).

## 18 Mechanisms of immune modulation

Dendritic cells represent primary targets for Al-CPI-mediated immunomodulation. Human monocyte-derived dendritic cells (moDCs) treated with Al-CPI demonstrate significant transcriptional changes, with 444 differentially expressed transcripts identified upon stimulation (24). The most prominent changes involve upregulation of genes encoding Kruppel-like factor 10 (KLF10) and low-density lipoprotein receptor (LDLR), along with multiple genes involved in cholesterol biosynthesis and sterol regulatory element-binding protein (SREBP) signaling pathways (24). The metabolic reprogramming induced by Al-CPI includes upregulation of key enzymes in the mevalonate pathway, including 3-hydroxy-3-methylglutaryl-CoA synthase 1 (HMGCS1), 3-hydroxy-3-methylglutaryl-CoA reductase (HMGCR), and mevalonate kinase (MVK). This metabolic shift toward cholesterol biosynthesis may represent a novel mechanism of immune modulation, as cholesterol regulatory pathways are known to influence dendritic cell maturation and their capacity to promote either immunogenic or tolerogenic responses (24). Functionally, Al-CPI treatment of dendritic cells results in reduced expression of co-stimulatory molecules CD86, CD83, and HLA-DR, even in the presence of lipopolysaccharide (LPS) stimulation (26). This suppression of maturation markers is accompanied by increased IL-10 production and decreased pro-inflammatory cytokine secretion, indicating a shift toward a more tolerogenic phenotype (27). Al-CPI exerts profound effects on T cell differentiation and function. In experimental models, treatment with Al-CPI significantly increases the frequency of regulatory T cells (Tregs) expressing CD4+CD25+Foxp3+ phenotype. This enhancement of regulatory T cell populations is associated with increased production of anti-inflammatory cytokines IL-10 and TGF-β, while simultaneously reducing Th2-associated cytokines IL-4, IL-5, and IL-13 (27).

The modulation of T cell responses appears to be partially dependent on IL-10 signaling, as treatment with anti-IL-10 receptor antibodies partially reverses some of the immunomodulatory effects of Al-CPI (27). However, the persistence of certain effects even after IL-10 receptor blockade suggests that multiple mechanisms contribute to the overall immunomodulatory activity of this helminth-derived molecule. Al-CPI treatment significantly alters humoral immune responses, particularly affecting immunoglobulin isotype switching. In mouse models, Al-CPI administration leads to substantial reduction in total and allergen-specific IgE production while promoting IgG2a responses (27). This shift in antibody isotype profiles is consistent with the promotion of Th1 and regulatory responses over Th2-mediated allergic reactions. Interestingly, despite inducing specific IgE responses in some individuals from endemic populations, Al-CPI does not elicit functional IgE-mediated reactions in basophil activation tests or histamine release assays. This dissociation between IgE binding and biological activity suggests that Al-CPI possesses low allergenic potential despite being recognized by the adaptive immune system (28).

**Figure 5:**
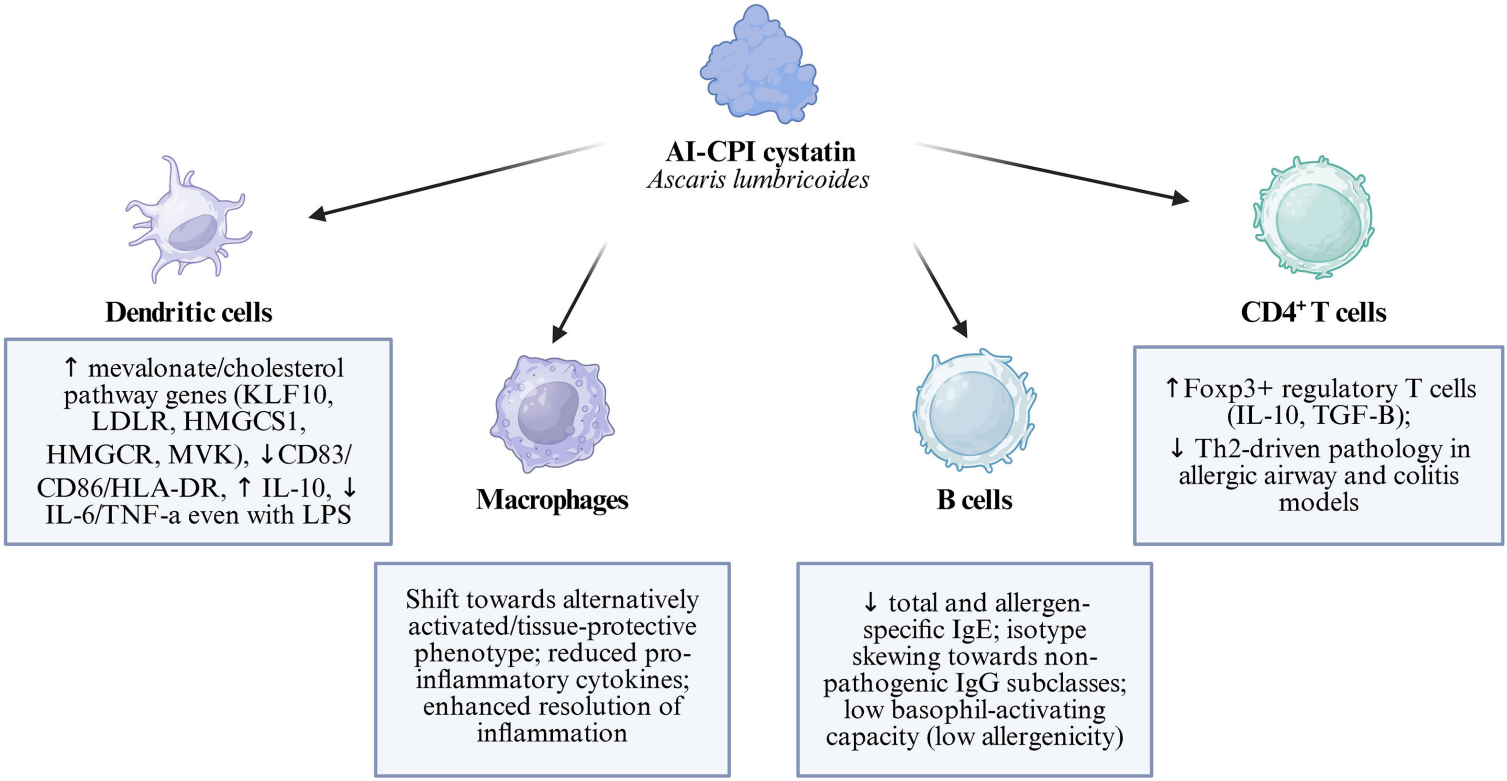
Al-CPI (Ascaris lumbricoides cystatin) immunomodulatory mechanisms.

**Figure 5** presents the integrated mechanism of Al-CPI, including dendritic cell metabolic reprogramming, suppression of co-stimulatory molecules, and induction of regulatory T cell responses that collectively promote immune tolerance.

## 19 Therapeutic applications in inflammatory diseases

The therapeutic potential of Al-CPI in inflammatory bowel disease has been demonstrated in experimental colitis models. In the DSS-induced colitis model, rAl-CPI demonstrated robust therapeutic effects when administered intraperitoneally at 0.5 µg/g or 0.25 µg/g body weight once daily for 14 days (seven days before and seven days during DSS exposure), resulting in significant improvements in disease activity index, myeloperoxidase activity, and histological scores. Similarly, in the allergic airway inflammation model, prophylactic intraperitoneal dosing of 20 µg per mouse (approximately 1 µg/g) on days 0, 7, 14, and 21—both before and during house dust mite sensitization—prevented airway hyperreactivity and eosinophilia. No studies have yet characterized rAl-CPI’s pharmacokinetics or pharmacodynamics, but preliminary data indicate the recombinant is highly thermostable and retains protease inhibitory activity after prolonged storage. Allergenicity assessments beyond basophil activation tests revealed no increase in histamine release or anaphylactic signs following rAl-CPI administration, confirming its low allergenic potential.

Treatment with recombinant Al-CPI significantly ameliorates dextran sodium sulfate (DSS)-induced colitis, as evidenced by reduced disease activity index, decreased myeloperoxidase activity, and improved histological scores (29). The protective effects are associated with overexpression of anti-inflammatory genes IL10 and TGFB, accompanied by reduced expression of pro-inflammatory mediators IL6 and TNFA.

The mechanism of protection in colitis models appears to involve multiple pathways. Al-CPI treatment induces alternatively activated macrophages characterized by high IL-10 production, which contributes to intestinal epithelium repair and resolution of inflammation (29). Additionally, the upregulation of TGF-β expression, a cytokine known for its role in maintaining intestinal homeostasis and promoting regulatory T cell development, further supports the therapeutic efficacy of Al-CPI in inflammatory bowel conditions (29).

Al-CPI demonstrates remarkable efficacy in preventing allergic airway inflammation in mouse models. Prophylactic treatment with Al-CPI before sensitization with house dust mite allergens significantly reduces airway hyperreactivity, eosinophil infiltration, and goblet cell hyperplasia. In mouse models of allergic airway inflammation, prophylactic administration of rAl-CPI before sensitization with Blomia tropicalis extract effectively prevented the development of disease features. Intraperitoneal dosing of 20 µg rAl-CPI per mouse on days 0, 7, 14, and 21—both before and during HDM sensitization—resulted in significant reductions in airway hyperreactivity to methacholine challenge, eosinophil and neutrophil infiltration in bronchoalveolar lavage, and goblet cell hyperplasia in lung tissue. These protective effects are accompanied by suppression of Th2 cytokines and promotion of regulatory immune responses (27). The anti-allergic properties of Al-CPI appear to be mediated through multiple mechanisms, including interference with antigen presentation by dendritic cells, promotion of regulatory T cell development, and direct suppression of effector cell activation (27). The ability to prevent rather than merely treat established allergic inflammation suggests that Al-CPI could be particularly valuable for individuals at high risk of developing allergic diseases.

Beyond inflammatory bowel disease and allergic asthma, the broad immunomodulatory properties of Al-CPI suggest potential applications in various other inflammatory conditions. The ability to suppress pro-inflammatory cytokine production while enhancing regulatory responses makes Al-CPI a candidate for treating autoimmune diseases, chronic inflammatory conditions, and potentially organ transplant rejection (8). Preliminary studies suggest that excretory/secretory products from A. suum, which include cystatin among other bioactive compounds, can improve outcomes in experimental colitis models and demonstrate conserved bioactive properties across multiple helminth species (26). These findings support the potential for developing helminth-derived therapeutics with broad anti-inflammatory applications.

## 20 Future perspectives and clinical translation

The translation of Al-CPI from experimental studies to clinical applications requires addressing several key challenges. First, the development of scalable production methods for pharmaceutical-grade recombinant Al-CPI will be essential for clinical testing (29). Second, determination of optimal dosing regimens and administration routes will require careful pharmacokinetic and pharmacodynamic studies. Third, although murine studies have revealed the generation of non-functional IgE against Al-CPI without allergic manifestations—a favorable finding for its safety profile—clinical translation will necessitate comprehensive immunogenicity assessments in humans to rule out unexpected immune responses.

The mechanism of action studies revealing effects on cholesterol metabolism and SREBP signaling pathways open new avenues for understanding helminth immunomodulation and potentially identifying biomarkers for therapeutic monitoring (24). These metabolic changes may also suggest combination therapeutic approaches that target both inflammatory pathways and metabolic dysfunction in chronic inflammatory diseases.

## 21 Brugia malayi

*Brugia malayi* is a tissue-dwelling filarial nematode and one of the primary causative agents of lymphatic filariasis, a neglected tropical disease that leads to considerable morbidity and socio-economic burden worldwide. Aside from its well-documented pathogenicity, *B. malayi* has been a source of biologically active molecules with profound immunomodulatory properties, which not only assist the parasite in evading host defenses but also present promising avenues for therapeutic interventions in immune-mediated diseases.

## 22 Molecular mechanisms of immune modulation

Both microfilariae (Mf) and Mf-derived EVs downregulate human dendritic cell (DC) activation and cytokine production, particularly reducing IL-12 and other pro-inflammatory mediators after stimulation with LPS and IFN-γ. EVs were revealed to alter gene expression in DCs, showing downregulation of antigen-processing molecules (e.g., HLA-DMA, HLA-DMB) and impairing Th1/Th2 activation pathways. This suppression consequently impairs antigen-specific CD4+ T cell responses, decreasing not only their activation (CD154) but also IFN-γ output, even against strong viral antigens like SARS-CoV-2 peptides (30). Mf- and EV-derived factors drive the expansion of IL-10-producing CD4+ T cells and regulatory monocytes/macrophages, thereby inducing a tolerant or hypo-responsive immune milieu in chronic filariasis. These products block the maturation of DCs, downregulating co-stimulatory and antigen-uptake receptors, impairing T cell activation and skewing immunity toward tolerance or Th2 dominance (30).

These modulatory effects were linked specifically to EVs, which were isolated from Mf E/S by ExoQuick-TC ULTRA precipitation and purified by ultracentrifugation at 100 000 × g for 1 hour to remove contaminating soluble proteins; EV preparations were characterized by enrichment of exosomal markers (e.g., CD9, CD63, TSG101) and judged free of soluble protein contaminants by protein assay of the depleted supernatant and of endotoxin (LPS) by Limulus amebocyte lysate testing.

Galectins are prominent ES proteins in filarial nematodes. *B. malayi* galectin-2 is a tandem-repeat-type galectin highly conserved among nematodes. It features two functional carbohydrate recognition domains (CRDs) and is released in significant quantities, particularly during the late stages of microfilarial development and by adult females. Recombinant BmLec-2 (rBmLec-2) binds mammalian galactoside glycans, exhibits hemagglutination activity (dependent on intact CRDs), and binds to several host immune glycoproteins, potentially mimicking or antagonizing human galectins (notably Gal-9). Its binding profile suggests it may modulate B cell, T cell, and mast cell functions (9). A significant proportion of humans with loiasis or bancroftian filariasis develop antibodies to rBmLec-2, highlighting exposure and immune recognition, although its diagnostic specificity is limited by cross-reactivity (9).

*B. malayi* cystatin is a potent protease inhibitor with defined immunoregulatory properties. Administration of recombinant cystatin (rBmCys) in animal models of rheumatoid arthritis attenuates disease severity by downregulating IFN-γ and TNF-α and enhancing IL-4/IL-10—promoting a shift toward an anti-inflammatory Th2 profile (10). Cystatins inhibit host proteases essential for antigen processing, thereby dampening T cell priming and shifting the cytokine milieu (10). rBm33, a pepsin inhibitor secreted by all life stages, modulates host PBMCs primarily by inducing Th1-skewed responses, high TNF-α, IL-6, and NO production. rBm33 stimulates MyD88/gene expression, and activates JNK/c-Fos pathways, indicating it invokes pro-inflammatory transcriptional programs without causing oxidative stress (31). ALT-2 is a major L3-stage antigen, essential for early host-parasite interactions and a promising vaccine candidate. ALT-2, especially as a fusion protein with tuftsin (which targets macrophage/dendritic cell uptake), enhances humoral and cellular responses, generates high titers of IgG1/IgG2a/IgG2b (Th1/Th2 balance), increases IFN-γ, IL-2, IL-5, IL-10, and demonstrates significant *in vitro* cytotoxicity against L3 larvae—indicating both direct and ADCC effector mechanisms (32). Administration of recombinant ALT-2 in murine models of type 1 diabetes leads to lower blood glucose, reduced islet damage, decreased IFN-γ/TNF-α, and increased IL-4, IL-5, IL-10 secretion, thus ameliorating autoimmunity through Th2 polarization and regulatory mechanisms (33). Calreticulin, a major secretory and surface protein, interferes with host complement activation. BmCRT directly binds human C1q at sites critical for IgG/IgM/CRP interaction, thus blocking classical complement activation at an early stage—preventing C1r2–C1s2 complex activation and downstream lysis (34, 35). Immunization with rBmCRT in mice and Mastomys coucha induces strong IgG1/IgG2a/IgG2b/IgG3 responses, elicits Th1 and Th2 cytokine secretion (notably, high IFN-γ and IL-10), and leads to marked reductions in microfilaria and adult worm loads in permissive infection models. BmCRT also strongly activates macrophages and increases their antigen uptake and effector functions (34).

BmIL5Rbp is a secreted molecule that antagonizes the human IL-5 receptor, inhibiting eosinophil activation and survival, thus blunting a key effector arm of host defense against helminths. BmIL5Rbp localizes to the parasite’s surface and excretory/secretory products, directly inhibiting IL-5 binding to its receptor. RNA interference-mediated knockdown of BmIL5Rbp in the parasite reduces its ability to inhibit IL-5-driven responses, demonstrating a unique example of a parasite-encoded cytokine antagonist (36).

## 23 Clinical and therapeutic applications

Several *B. malayi* proteins, notably ALT-2, cystatin, pepsin inhibitor, and calreticulin, have been evaluated for use in vaccine formulations and as immunomodulatory agents in experimental models. ALT-2, Cystatin, and Calreticulin when delivered as recombinant proteins or in fusion/novel adjuvant forms (e.g., with tuftsin or liposomes), induce robust humoral (IgG, IgG subclasses) and cellular (IFN-γ, IL-10, IL-4) immune responses, demonstrating protection in murine and rodent infection models and efficacy in reducing worm burdens or attenuating autoimmune pathology (e.g., type 1 diabetes, rheumatoid arthritis) (10, 32–34, 37). Cystatin and ALT-2 both show efficacy in suppressing experimental arthritis and type 1 diabetes, reflecting their capacity to reset Th1/Th2 balance, reduce pro-inflammatory cytokines, and enhance regulatory cytokine production without the broad immunosuppression typical of conventional anti-inflammatory agents (10, 33). BmIL5Rbp and Galectin-2 - parasite-encoded antagonists and molecular mimics offer templates for designing human cytokine or glycan pathway modulators. BmIL5Rbp’s inhibition of eosinophil activation could inspire new therapies for hyper-eosinophilic syndromes or allergy modulation (9, 36).

Understanding the structural basis of BmCRT–C1q interaction could facilitate the development of highly selective complement inhibitors for diseases driven by aberrant classical pathway activation (34, 35).

The use of liposomes to deliver *B. malayi* antigens multiplies the antigen-specific antibody titers and delivers long-lived, quantitatively superior immune responses—a model for optimizing vaccine design against helminths and potentially other chronic pathogens (37).

## 24 Fasciola hepatica

Commonly known as the liver fluke, *Fasciola hepatica* is a globally significant trematode parasite causing fasciolosis in both livestock and humans. As an emerging zoonotic disease, it represents a considerable public health burden in endemic regions (38). The parasite’s complex life cycle involves migration through intestinal and hepatic tissues, during which it releases a sophisticated array of excretory-secretory products (ESPs) that actively reshape the host immune environment to ensure long-term survival (39). Research into *F. hepatica* has revealed not only key mechanisms of helminth-driven immunomodulation but also the potential therapeutic utility of its immunoregulatory molecules in inflammatory and autoimmune disorders.

## 25 Immunomodulatory mechanisms

*F. hepatica* employs multiple strategies to suppress pro-inflammatory responses and promote an anti-inflammatory or regulatory milieu. Among its ESPs, several well-characterized molecules target innate immune cells, particularly dendritic cells (DCs) and macrophages. The cysteine protease cathepsin L3 (FhCL3), expressed predominantly in the invasive juvenile stage, triggers a non-canonical activation of the NLRP3 inflammasome in murine DCs, leading to IL-1β and IL-18 secretion independently of caspase-1 and without requiring prior microbial priming (38). This alternative inflammasome activation depends on the enzymatic activity of FhCL3, involves reactive oxygen species (ROS) production, K^+^ efflux and lysosomal acidification, and conditions DCs to prime T cells toward a mixed IFN-γ and IL-13 profile—a response that may influence parasite control or chronicity (38).

**Figure 6:**
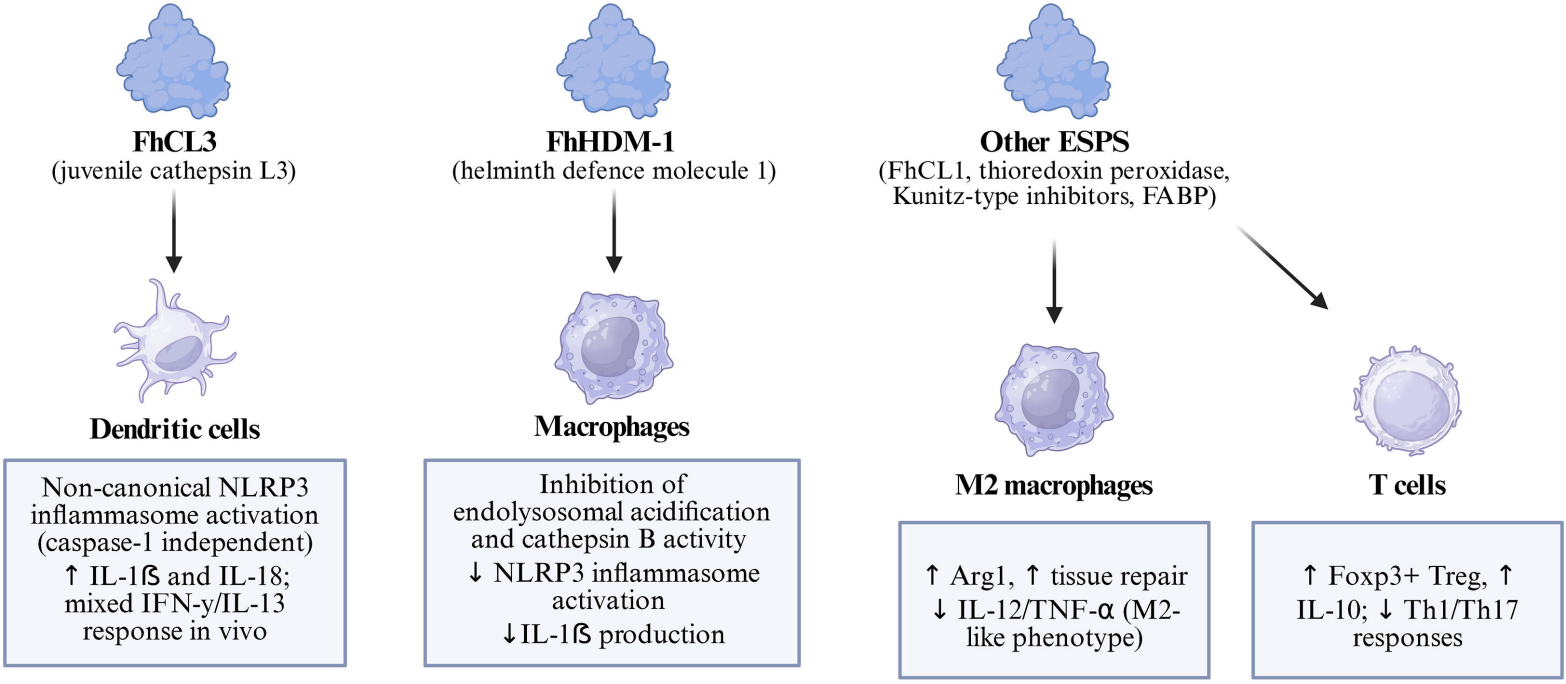
Immunomodulatory mechanisms of F. hepatica.

Conversely, other parasite products actively inhibit inflammatory pathways. The helminth defense molecule-1 (FhHDM-1), a cathelicidin-like peptide secreted across all life-cycle stages, prevents NLRP3 inflammasome activation by inhibiting endolysosomal acidification in macrophages, thereby blocking cathepsin B activation and IL-1β maturation (40). This suppression of IL-1β helps to maintain a Th2-favorable environment, which supports parasite persistence. Additionally, molecules such as FhCL1, thioredoxin peroxidase, Kunitz-type protease inhibitors, and fatty-acid binding proteins modulate DC and macrophage functions, often through TLR4 interference or the induction of alternative (M2) activation, resulting in downregulation of IL-12, TNF and other Th1-promoting cytokines (39). Together, these mechanisms collectively dampen Th1/Th17 responses while fostering regulatory T-cell development and tissue-repair programs, allowing the parasite to mitigate host damage and establish chronic infection. **Figure 6** summarizes the dual immunomodulatory strategies of F. hepatica, including activation of non-canonical inflammasome pathways by FhCL3 and inhibition of inflammatory signaling by FhHDM1, resulting in a regulatory immune environment.

## 26 Clinical applications

The potent and multifaceted immunoregulatory properties of *F. hepatica* ESPs have spurred interest in their therapeutic potential for immune-mediated disorders. The ability of FhHDM-1 to specifically inhibit NLRP3 inflammasome activation positions it as a candidate for treating diseases driven by excessive IL-1β, such as rheumatoid arthritis, gout, or metabolic syndromes (40). Similarly, FhCL3’s unique capacity to induce an alternative inflammasome activation and a balanced IFN-γ/IL-13 T-cell response could be harnessed to modulate immune polarization in allergic or autoimmune settings (38). Moreover, whole parasite extracts or defined ESPs (e.g**.,** Kunitz-type molecule, Fh12) have shown efficacy in preclinical models of collagen-induced arthritis, where they suppress inflammation and promote Treg expansion (39). These findings underscore the potential of *F. hepatica*-derived immunomodulators as novel biologics or as templates for designing synthetic anti-inflammatory agents, offering a promising avenue for helminth-inspired therapeutics in autoimmunity and chronic inflammation.

## 27 Conclusion

Helminth-derived immunomodulators exemplify how long-term host–parasite co-evolution has yielded highly specialized mechanisms capable of recalibrating dysregulated immunity with therapeutic precision. ES-62 illustrates this paradigm: by selectively routing key inflammatory signaling components to autophagolysosomal degradation, it restores immune homeostasis rather than broadly suppressing responses, and its translation into synthetic small-molecule analogues demonstrates the feasibility of harnessing parasite strategies for human anti-inflammatory drug development. Across species, convergent themes emerge. Ancylostoma duodenale deploys a coordinated arsenal—protease inhibitors, anticoagulants, anti-inflammatory proteins, and tissue-modifying enzymes—to establish chronic, low-pathology infections, revealing pathways that could be repurposed for treating inflammatory, autoimmune, and hemostatic disorders. Anisakis pegreffii adds nuance through context-dependent modulation, inducing dendritic cell tolerization, macrophage polarization, and regulatory T-cell expansion while exhibiting temperature- and tissue-specific antigen expression; these features underscore both the therapeutic promise of derived compounds and the need to manage strain variability, dose-dependent toxicity, and dual pro-/anti-inflammatory potential. Ascaris lumbricoides cystatin (Al-CPI) offers a compelling clinical candidate by reshaping dendritic cell function, expanding Tregs, dampening maladaptive Th2 responses, and reprogramming cellular metabolism—including cholesterol biosynthesis—resulting in robust efficacy across experimental models of allergic asthma and inflammatory bowel disease with a favorable safety and allergenicity profile. Brugia malayi further broadens the toolkit with a diverse secretome that redirects host immunity and shows activity beyond helminth infection, informing vaccine adjuvants and immunotherapies through precise pathway targeting and host–protein interactions. Collectively, these systems demonstrate that targeted immune rebalancing—rather than blanket immunosuppression—is achievable via well-defined molecular interventions. While helminth-derived molecules primarily act as immunomodulators to restore homeostasis, they can also exhibit dual roles with potential harmful effects. For instance, excessive immunosuppression may exacerbate bacterial or viral co-infections by impairing neutrophil recruitment and antibody responses, leading to higher pathogen loads (41, 42). In untreated infections, these molecules contribute to malnutrition, anemia, and tissue damage. This duality underscores the need for careful modality selection to harness benefits while minimizing risks in therapeutic applications.

The path forward centers on isolating and characterizing specific effector molecules; decoding cell-type, pathway, and biophysical dependencies; engineering stable, scalable biologics or small-molecule mimetics; and rigorously evaluating safety to avoid pathogenic sequelae. Translation faces significant methodological challenges. Current evidence relies predominantly on *in vitro* and animal models, with limited clinical validation. Manufacturing complexities, immunogenicity concerns, endotoxin contamination, and regulatory hurdles represent substantial barriers. For complex molecules like ES-62 and filarial EVs, rigorous purification protocols and contamination controls are essential. Small-molecule analogues may offer advantages in scalability and regulatory approval, but require extensive structure-activity relationship studies to maintain biological activity while ensuring safety. As genomic and proteomic mapping accelerates target discovery, helminth-inspired agents are poised to inaugurate a new class of precision immunomodulators with broad utility against chronic inflammatory and autoimmune diseases.

## Glossary

ABP: Anisakis body proteins
ADCC: Antibody-dependent cell-mediated cytotoxicity
AduNAP4: Ancylostoma duodenale nematode anticoagulant peptide 4
AduTIL-1: Ancylostoma duodenale trypsin inhibitor-like 1
AIP-1/AIP-2: Anti-Inflammatory Protein 1/2
Al-CPI: Ascaris lumbricoides cysteine protease inhibitor
ALT-2: Abundant Larval Transcript Protein-2
ANOVA: Analysis of Variance
Arg-1: Arginase-1
BCR: B cell receptor
BmCRT: Brugia malayi calreticulin
BmIL5Rbp: Brugia malayi IL-5 receptor binding protein
C1q: Complement component 1q
CCL3/CCL4: C-C motif chemokine ligand 3/4
Ccl2: C-C motif chemokine ligand 2
CCR7: C-C chemokine receptor type 7
CD4+: Cluster of differentiation 4 positive
CD8+: Cluster of differentiation 8 positive
CD25+: Cluster of differentiation 25 positive
CD83: Cluster of differentiation 83
CD86: Cluster of differentiation 86
CD154: Cluster of differentiation 154
CE: Crude extract
CIA: Collagen-induced arthritis
CRDs: Carbohydrate recognition domains
CRP: C-reactive protein
CXCL10: C-X-C motif chemokine ligand 10
DC: Dendritic cells
DNase-II: Deoxyribonuclease II
DSS: Dextran sodium sulfate
ERK1/2: Extracellular signal-regulated kinase 1/2
ES: Excretory-secretory
ES-62: Excretory-secretory glycoprotein 62
EVs: Extracellular vesicles
FcϵRI: High-affinity IgE receptor
Foxp3+: Forkhead box P3 positive
H3c7: Histone cluster 3 H3 family member c7
HLA-DR: Human leukocyte antigen-DR
HLA-DMA/HLA-DMB: Human leukocyte antigen-DMA/DMB
HMGCR: 3-Hydroxy-3-methylglutaryl-CoA reductase
HMGCS1: 3-Hydroxy-3-methylglutaryl-CoA synthase 1
IFN-γ: Interferon gamma
Igα/β: Immunoglobulin alpha/beta
IgE: Immunoglobulin E
IgG: Immunoglobulin G
IgG1/IgG2a/IgG2b/IgG3: Immunoglobulin G subclasses
IgM: Immunoglobulin M
IL-1α: Interleukin-1 alpha
IL-1β: Interleukin-1 beta
IL-2: Interleukin-2
IL-4: Interleukin-4
IL-5: Interleukin-5
IL-6: Interleukin-6
IL-10: Interleukin-10
IL-12: Interleukin-12
IL-12p40/p70: Interleukin-12 subunits
IL-13: Interleukin-13
IL-17: Interleukin-17
IL-22: Interleukin-22
IL-23: Interleukin-23
JNK: c-Jun N-terminal kinase
Ki: Inhibition constant
KLF10: Kruppel-like factor 10
L3: Larval stage 3
LC3: Microtubule-associated protein 1 light chain 3
LDLR: Low-density lipoprotein receptor
Lec-2: Galectin-2
LPS: Lipopolysaccharide
Mf: Microfilariae
MHC-II: Major histocompatibility complex class II
moDCs: Monocyte-derived dendritic cells
MVK: Mevalonate kinase
MyD88: Myeloid differentiation primary response 88
NETosis: Neutrophil extracellular trap formation/osis
NETs: Neutrophil extracellular traps
NF-κB: Nuclear factor kappa B
NO: Nitric oxide
p62: Sequestosome 1
PBMCs: Peripheral blood mononuclear cells
PC: Phosphorylcholine
PI 3-kinase: Phosphoinositide 3-kinase
PKC: Protein kinase C
PKC-α: Protein kinase C-alpha
PKCδ: Protein kinase C-delta
PLD: Phospholipase D
rBm33: Recombinant Brugia malayi pepsin inhibitor 33
rBmCys: Recombinant Brugia malayi cystatin
rBmLec-2: Recombinant Brugia malayi galectin-2
ROS: Reactive oxygen species
SDS-PAGE: Sodium dodecyl sulfate-polyacrylamide gel electrophoresis
sICAM: Soluble intercellular adhesion molecule
SMA: Small-molecule analogues
SPH K-1: Sphingosine kinase-1
SREBP: Sterol regulatory element-binding protein
TCR: T cell receptor
TGF-β: Transforming growth factor beta
Th1: T helper 1
Th2: T helper 2
TIL: Trypsin inhibitor-like
TLR: Toll-like receptor
TLR2: Toll-like receptor 2
TLR4: Toll-like receptor 4
TLR9: Toll-like receptor 9
TNF-α: Tumor necrosis factor alpha
Treg: Regulatory T cells.
